# *Wolbachia* Induces Structural Defects Harmful to *Drosophila simulans* Riverside Spermiogenesis

**DOI:** 10.3390/cells12192337

**Published:** 2023-09-22

**Authors:** Maria Giovanna Riparbelli, Ambra Pratelli, Giuliano Callaini

**Affiliations:** Department of Life Sciences, University of Siena, Via Aldo Moro 2, 53100 Siena, Italy; riparbelli@unisi.it (M.G.R.); pratelli3@student.unisi.it (A.P.)

**Keywords:** *Wolbachia*, *Drosophila simulans*, spermiogenesis, sperm defects, ultrastructure

## Abstract

The relationship between cytoplasmic incompatibility and the obligate intracellular alphaproteobacteria *Wolbachia* has for a long time been reported. Although the molecular mechanisms responsible for this reproductive alteration are beginning to be understood, the effects of *Wolbachia* on germ cell structure and dynamics have not yet been fully investigated. We report here that the presence of *Wolbachia* in infected cysts of elongating spermatids is associated with major structural defects that become more evident in mature sperm. We find mitochondrial defects, an improper axoneme structure, reduced sperm numbers, and individualization failures. The large heterogeneous variety of the ultrastructural defects found in elongating spermatids and mature sperm provide the first cytological evidence for the reduced fertility associated with *Wolbachia* infection in *Drosophila simulans* males. The observed abnormalities could be the result of the mechanical stress induced by the high bacteria numbers during the process of spermatid elongation, rather than the result of the released factors affecting the proper morphogenesis of the germ cells. Moreover, high *Wolbachia* densities in male germ cells may not be appropriate for causing cytoplasmic incompatibility as the bacteria are harmful for spermatid differentiation, leading to abnormal sperm that is unlikely to be functional.

## 1. Introduction

A large number of arthropods and some nematodes harbour maternally inherited obligate intracellular alphaproteobacteria belonging to the genus *Wolbachia*. These bacteria that inhabit the somatic and germinal tissues of their hosts have attracted considerable interest because of their mutual interaction with filarial nematodes [1] and their ability to reduce the spread of pathogenic arboviruses, including dengue, *Zika*, chikungunya, and yellow fewer, hosted in some insect vectors [2,3,4]. It has been demonstrated, indeed, that filaria do not survive in the absence of essential metabolites supplied by *Wolbachia*, suggesting that antibiotic treatment could represent a promising successful tool for controlling human filariasis [5]. On the other hand, *Wolbachia* infection in mosquitoes reduces or prevents the transmission of insect-borne diseases, affecting the virus life cycle [6], by competing for essential cellular metabolic resources needed for both bacteria and virus replication [7,8]. 

*Wolbachia* is the most widespread heritable intracellular bacterium found in arthropods [9,10,11], where they manipulate the reproduction of their hosts, causing a plethora of reproductive alterations, including feminization, parthenogenesis, male killing, and cytoplasmic incompatibility [12]. 

Cytoplasmic incompatibility (CI) is the most common reproductive alteration found in insects and occurs when *Wolbachia*-infected males mate with uninfected females. This unidirectional cross leads, in *Drosophila simulans*, to the abnormal compaction of the paternal chromatin and to its defective segregation during the first zygotic division, resulting in early embryo death [13,14]. Remarkably, CI is rescued in *Wolbachia*-infected females, giving a fitness advantage to the infected populations that promote the diffusion of the bacteria [15]. It has been demonstrated, indeed, that infected strains of *Drosophila simulans* progressively replace, in California, the uninfected populations [16]. 

The spreading advantage of the infected populations over the uninfected ones has, however, some fitness costs due to the general tropism of *Wolbachia* for somatic tissues and germ cells [17]. For example, the presence of *Wolbachia* in the *Drosophila* brain [18] may play a role in modulating host behaviour [19,20], whereas a high bacteria density in somatic tissues can lead to a reduced lifespan in flies [21]. Moreover, *Wolbachia*-bearing *Drosophila simulans* males have reduced fertility and transfer fewer sperm than uninfected males [22,23]. *Wolbachia* infection is also associated with a reduced rate in sperm competition [24].

Although the molecular mechanism of the CI is beginning to be understood with the discovery of the *Wolbachia* prophage WO genes *cifA* and *cifB* [25,26,27,28], the fertility problems and the reduced sperm production in infected males are still unclear. 

It is unlikely that the fertilization problems are due to the physical presence of *Wolbachia* in mature sperm, since the bacteria are stripped to the distal waste bag of the elongated spermatids by the actin cones during the individualization process [29]. Rather, the fact that the bacteria inhabit in high numbers almost all the spermatocytes and early spermatids [29,30] raises the question of whether their density can interfere with the proper dynamics of the germ’s 64 cells. For example, can a high bacterial density be compatible with the proper assembly of the meiotic spindles and with the correct germ cell division? Can the bacteria influence the elongation of the early spermatids and the movement of the individualization complexes?

Cysts of sperm with axoneme pairs have been reported in *Wolbachia*-infected *Drosophila simulans*, likely due to the failure of the second meiosis [29]. Moreover, *Wolbachia* infection has been associated in *Drosophila melanogaster* to the defective shaping of mitochondrial derivatives during spermatid elongation [31]. However, such minor abnormalities do not explain the reduced number of mature sperm produced by infected males.

We hypothesized that the high bacteria density observed in the male germ cells of infected flies could cause dramatic structural defects to the elongating spermatids, altering their proper dynamics. To verify this possibility, we performed a careful ultrastructural analysis of the spermiogenesis in *Drosophila simulans* to understand the impact of the distribution of *Wolbachia* on sperm maturation. 

We describe here the abnormalities commonly found in infected testes. It is very difficult to report a confident percentage of abnormal cysts per testis by electron microscopic analysis unless serial sections are made along the entire length of the spermatids. However, we found that approximately 12% of spermatid cysts were apparently normal (*n* = 359 cysts examined from random sections of 17 infected testes). 

Our results show that germ cells filled with high numbers of bacteria display several abnormal morphologies. We find cysts with reduced numbers of sperm, sperm with shorter or damaged axonemes, and mature sperm cysts with large cytoplasmic remnants. These heterogenic abnormalities could be the result of a mechanical stress induced by the overall occupancy of high numbers of bacteria within the germ cell cytoplasm that prevent a correct morphogenesis, rather than an indirect role of factors released by *Wolbachia*. All of the above structural defects can lead to the reduced sperm function and reduced fertility of infected males.

## 2. Materials and Methods

### 2.1. Flies

The *Wolbachia*-infected strain of *Drosophila simulans* was originally collected in Riverside CA, (DSR) and obtained from Dr. Michael Turelli. This *Drosophila* strain has high levels of bacterial infection and expression of cytoplasmic incompatibility. The control was the uninfected *Drosophila simulans* watsonville (DSW) strain kindly provided by Dr. Rosanna Giordano. Flies were grown on standard *Drosophila* medium in 200 mL plastic containers in a 12/12 light/dark cycle at 24 °C. 

It is possible to analyze spermiogenesis in both pupae and young males. However, the early stages of this process, which mainly consist of spermatid elongation, axoneme growth, and mitochondrial dynamics, are readily observable in mid-aged and old pupae. On the contrary, the individualization process and mature sperm are found in adults.

*Wolbachia* is absent in the DSW strain and we were unable to find the abnormalities observed in the infected DSR strain. Spermiogenesis and individualization process are normal in DSW males, resulting in cysts of 64 normal sperm. 

### 2.2. Transmission Electron Microscopy

Testes from mid-aged pupae (recognized by the yellow colour of the eyes) and late pupae (based on the appearance of wing and leg primordia) and 3–4 day old adults were dissected in phosphate-buffered saline (PBS), and fixed in 2.5% glutaraldehyde in PBS overnight at 4 °C. After rinsing for 30 min in PBS, the samples were post-fixed in 1% osmium tetroxide in PBS for 1 h. The samples were dehydrated in a graded series of ethanol, and then infiltrated with a mixture of Epon–Araldite resin and polymerized at 60 °C for 48 h. Ultrathin sections (50–60 nm thick) were cut with a LKB Ultratome NOVA, equipped with a diamond knife. The sections were collected with copper slot grids coated with formvar (1% in chloroform). After drying with filter paper, the sections were stained with 2% aqueous uranyl acetate for 20 min in the dark, and then with lead citrate for 2 min. The preparations were observed with a Tecnai G2 Spirit EM (FEI Eindhoven, The Netherlands) equipped with a Morada CCD camera (Olympus, Tokyo, Japan).

## 3. Results

Spermatogenesis in *Drosophila* starts at the apical end of the testis where a cluster of small postmitotic cells, the hub, release specific stemness signals to 8–10 surrounding germ stem cells and flanking somatic cyst stem cells [32]. The germ stem cells divide asymmetrically to self-renew and give an origin to differentiating cystoblasts that undergo four incomplete spermatogonial mitotic divisions to form cysts of 16 interconnected spermatocytes that undergo two successive meiotic divisions [29]. Spermatogenesis ends with the formation of cysts of 64 spermatids that undergo spermiogenesis. Spermiogenesis is a complex process which transforms round immotile spermatids in thin elongated moving sperm by a dramatic reorganization of the cytoplasmic organelles. The basal body, inherited by the cilium-like regions of the primary spermatocytes, nucleates the axoneme that acquires the dynein arms for sperm motion [33]. Concurrently to the elongation of the spermatid, the nucleus compacts, assuming a needle-like shape due to the gradual replacement of histones with protamines [34]. 

Given that sperm are highly specialized cells that undergo dramatic structural and morphological changes during spermiogenesis [35], it is conceivable that the eventual overproliferation of the bacteria within the germ cell cysts could induce obvious alterations of the germ cell dynamics. To better understand the effect of *Wolbachia* on the process of spermiogenesis, we carefully analyzed, by electron microscope, the testes of mid-aged and late pupae and young males from the infected *DSR* strain. 

### 3.1. Spermiogenesis in DSW Flies

To exclude the possibility that the abnormalities observed during the spermiogenesis of DSR could maybe be due to the internal host condition of the strain, rather than the presence of Wolbachia, we also analyze the spermiogenesis in the DSW strain that is naturally devoid of bacteria. 

**Figure 1:**
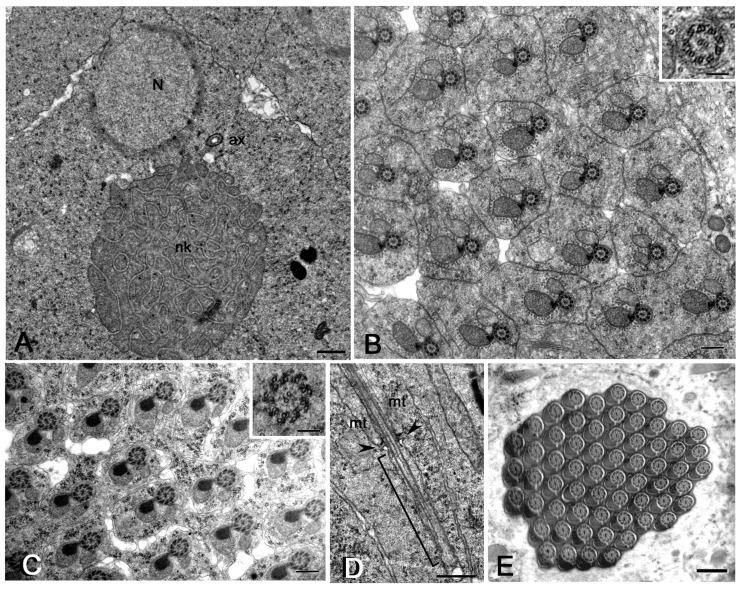
Spermiogenesis in *Drosophila simulans watsonville*. (**A**) Young spermatids: N, nucleus; nk, nebenkern; ax, axoneme. Cross sections of early (**B**) and mid-elongating spermatids (**C**) showing the uniform orientation of the axonemal/mitochondrial derivatives within the same cyst; details of the respective axonemes are shown in insets C and D. (**D**) Longitudinal section of an elongating spermatid showing the distal region of the growing axoneme enclosed within the ciliary cap (bracket); arrowheads point to the transition region between the cytoplasm and the ciliary cap; mt, mitochondria. (**E**) Cross-section of a mature sperm cyst; note the orderly disposition of the sperm. Scale bars: (**A**), 700 nm; (**B**,**C**), 400 μm; (**D**,**E**), 500 nm; insets, 100 nm.

Young spermatids at the beginning of the orientation, or onion stage, were characterized by round nuclei and distinct nebenkern (Figure 1A). The axoneme of early-elongating (Figure 1B) and mid-elongating (Figure 1C) spermatids consists of nine peripheral microtubule doublets, a cental tubule pair, and a remnant of the C-tubule (Figure 1B). The axoneme acquired nine peripheral tubules during spermatid elongation (Figure 1C). The major mitochondrial derivatives were darker in early-elongating spermatids (Figure 1B) and filled with dense material in mid-elongating spermatids (Figure 1C). Remarkably, the orientation of the spermatids within the same cysts was extremely regular during the spermiogenesis of DSW (Figure 1B,D). Never did we find bacteria within the elongating spermatid cysts of the uninfected DSW strain examined by cross-section (*n* = 97 cysts). 

The tip of the growing axoneme is associated in uninfected testes with a cap-like membranous structure, the ciliary cap, that extends approximately 4–5 μm over the posterior end of all the elongating spermatids examined from DSW (Figure 1D).

The process of spermiogenesis ends in DSW with the formation of cysts of 64 sperm in which the germ cells were tightly packed and regularly arranged in the same orientation (Figure 1E). No structural or numerical defects of the sperm or individualization abnormalities were observed.

### 3.2. Abnormal Mitochondria in Elongating Spermatids of Mid-Aged DSR Pupae

**Figure 2:**
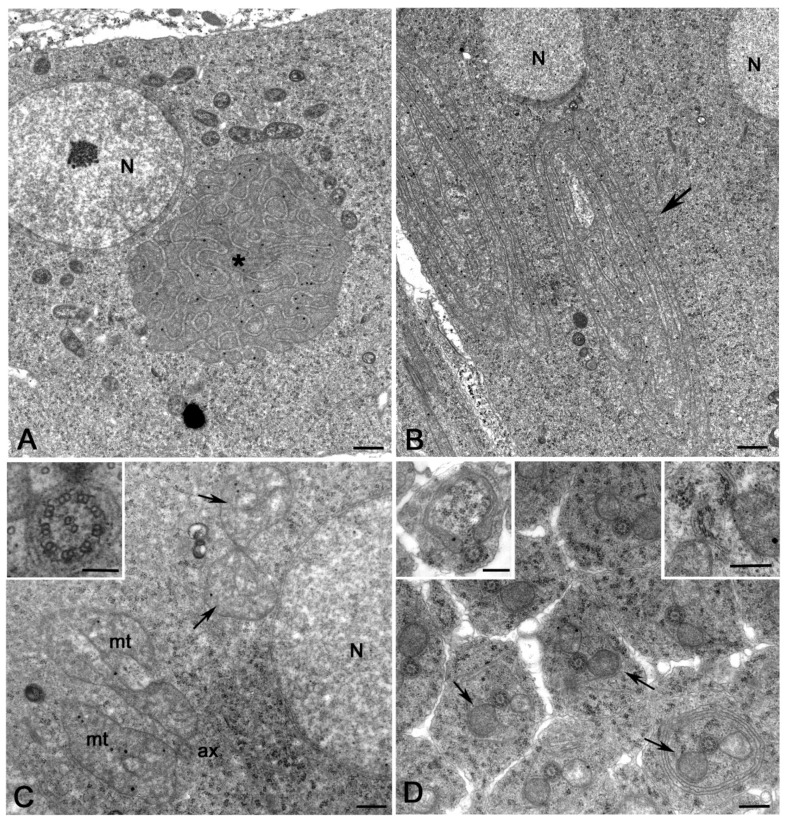
Mitochondria dynamics in young spermatids of mid-aged DSR pupae. (**A**) The small mitochondria fuse together to form a round onion-shape cluster (asterisk), the nebenkern; bacteria surround the nebenkern. (**B**) The nebenkern then unrolled (arrow) and resolved in two large structures that gradually elongate. (**C**) Spermatid at the beginning of elongation: each axoneme (ax) is usually located between two large mitochondria (mt) derived by the unfolding of the nebenkern; pairs of mitochondria lacking axonemes (arrows) are also found at this stage. The axoneme consists of nine peripheral doublets, one central tubule pair, and the peripheral C-remnant associated to the B-tubule (inset). (**D**) Spermatids at a more advanced elongation stage: the mitochondria pairs reduce in size and one of them becomes darker (arrows); abnormal mitochondria (left inset) or fragmented axonemes (right inset) are also found. N, nucleus. Scale bars: (**A**,**B**), 700 nm; (**C**,**D**), 500 nm; inset (**C**), 100 nm; left inset (**D**), 500 nm; right inset (**D**), 200 nm.

The small mitochondria scattered within the cytoplasm of the young spermatid fuse together to form a single round multilayered onion-shape nebenkern (Figure 2A). Bacteria surrounded the wrapped mitochondria but did not enter the nebenkern (Figure 2A). The nebenkern then unrolled in two large mitochondrial derivatives (Figure 2B). 

*Wolbachia* has never been enclosed within the mitochondria clusters during their transformation. The two growing mitochondrial derivatives were associated with the elongating axoneme (Figure 2C) that showed the conventional 9 + 2 model. The axonemes consisted, indeed, of the nine peripheral A- and B-tubules, the C-tubule remnant, and the central tubule pair (Figure 2C, inset). Cysts that are infected display some structural heterogeneity. Mitochondrial pairs lacking axonemes were also occasionally observed at this stage of development (12% of spermatids, *n* = 213 spermatids examined from five germ cell cysts) (Figure 2C). As the elongation of the spermatids progressed, the mitochondrial derivatives reduced in size and the larger one became darker (Figure 2D). Some young spermatids displayed abnormally shaped mitochondria (9% of spermatids, *n* = 178 spermatids examined from six germ cell cysts) (Figure 2D, left inset) or fragmented axonemes (5% of spermatids, *n* = 178 spermatids examined from six germ cell cysts) (Figure 2D, right inset).

**Figure 3:**
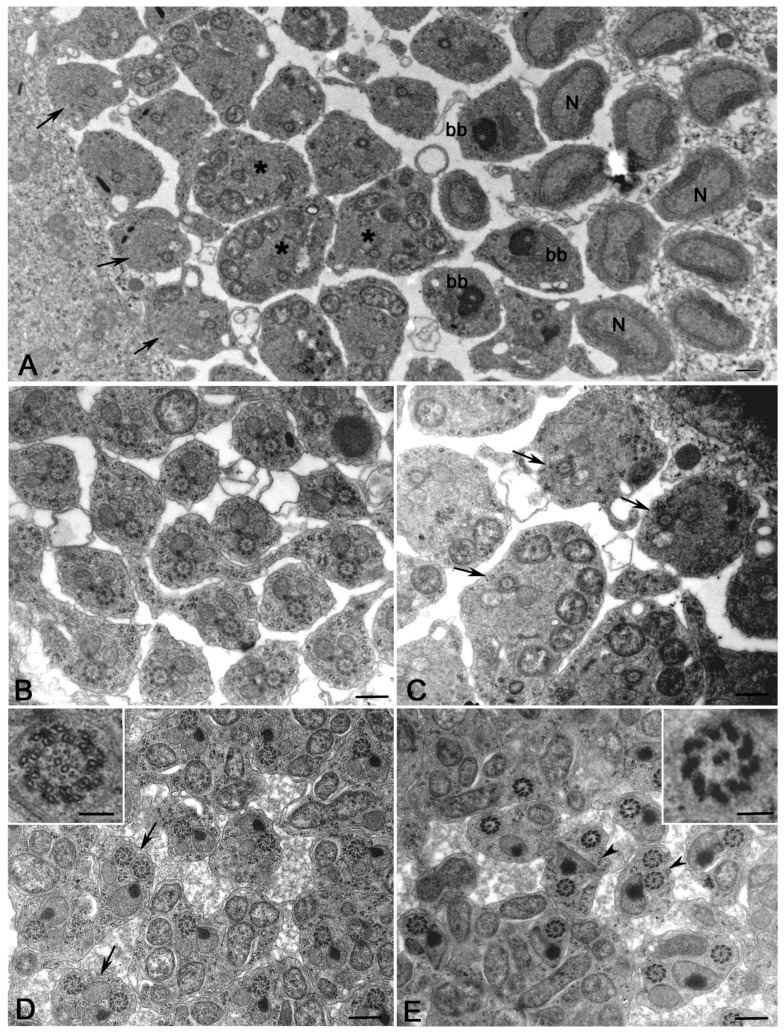
Elongating spermatids in testes of mid-aged DSR pupae. (**A**) Low magnification of the apical region of a cyst of young elongating spermatids showing the uneven disposition of the germ cells: in the same cross-section, the mid region (N) and the posterior region of the nuclei with the basal body (bb) are visible; spermatids with many bacteria (asterisks) and spermatids with few or no bacteria (arrows) are also evident. Early-elongating spermatids devoid of bacteria or with occasional bacteria have a reduced diameter, (**B**) whereas spermatids filled by bacteria (**C**) have a larger diameter; the complexes’ axoneme/mitochondria (arrows) are often displaced at the periphery of the infected cells. (**D**) The mitochondrial derivatives become more distinct in mid-elongating spermatids and the major one accumulates a cluster of dense material in front of the axoneme; spermatids with two axonemes and two mitochondria derivatives (arrows) are often found. The axoneme acquires nine peripheral tubules at this stage (inset). (**E**) Cross-section of mid-elongating spermatids in which most of the peripheral tubules, the central pairs, and the accessory fibers are filled by dense material that hide the structure of the axoneme (inset); spermatids with two axonemes and only one major mitochondrial derivative are also found (arrowheads). Scale bars: (**A–E**), 400 nm; insets, 100 nm.

Cross-sections of the apical region of early-elongating spermatids showed that the nuclei were misaligned (Figure 3A). Therefore, in the same section, we find spermatids at slightly different levels. Bacteria were numerous just posterior to the nuclei where the spermatids had a large cytoplasm, whereas they were rarely encountered further from the nuclei where the spermatids appeared thinner.

We, indeed, find several cysts consisting of spermatids of different dimension (29 of 37 cysts examined). Within these cysts, we scored thin spermatids apparently devoid of bacteria or with very few bacteria (63% of spermatids, *n* = 351 spermatids examined) (Figure 3B) and larger spermatids filled with many bacteria (37% of spermatids, *n* = 351 spermatids examined) (Figure 3C). Axonemes and mitochondria were often displaced to the periphery of the infected spermatids (Figure 3C). The absence of *Wolbachia* in early-elongating spermatids could also be explained by the uneven distribution of the bacteria during the early stages of sperm development. It has been shown by immunofluorescence observations of early spermatids that the bacteria are most abundant just posterior to the spermatid nucleus and populate the distal end of the tail in large numbers [29]. Thus, *Wolbachia* was hardly found in the middle region of spermatids. 

The two mitochondrial derivatives became more distinct in mid-elongating spermatids: the minor derivative further reduced in size while the major derivative accumulated a clump of paracrystalline material in front of the axoneme (Figure 3D). The axoneme acquired nine peripheral tubules (Figure 3D, inset), the so-called accessory fibers, that assembled by the modification of the C-tubule remnant. The infected and partially infected spermatid cysts examined at this stage of development (*n* = 27) showed a variable organization of the spermatid housed in the same cyst. We noticed that 67% of the spermatids scored (*n* = 672) usually consist of one axoneme and two mitochondrial derivatives. Conversely, 19% of the spermatids examined showed two axonemes and two mitochondria and 14% of the spermatids two axonemes and only one mitochondrion (Figure 3D,E).

This variability does not appear to correlate with the presence of *Wolbachia*, as 12% of the abnormal spermatids do not contain bacteria, based on randomly performed cross-sections. However, the presence of *Wolbachia* cannot be ruled out unless serial sections are made along the entire length of the spermatids.

Although, the mitochondrial derivatives elongated alongside and in close association with the growing axoneme (Figure 3D), we also observed spermatids in which the mitochondria have lost this continuity (21% of spermatids, *n* = 296 spermatids examined from five germ cell cysts) (Figure 3E). Moreover, there were spermatids in which the shape and size of the mitochondria were abnormal and spermatids in which the minor derivative was missing (23% of spermatids, *n* = 306 spermatids examined from seven germ cell cysts) (Figure 3E). Several cysts (19% of cysts, *n* = 37 germ cell cysts examined) contained mid-elongating spermatids that had axonemes in which the accessory fibers and often the peripheral tubules were filled with dense material, making their detection difficult (Figure 3E).

### 3.3. Structural Defects of the Growing Axonemes of Late DSR Pupae

**Figure 4:**
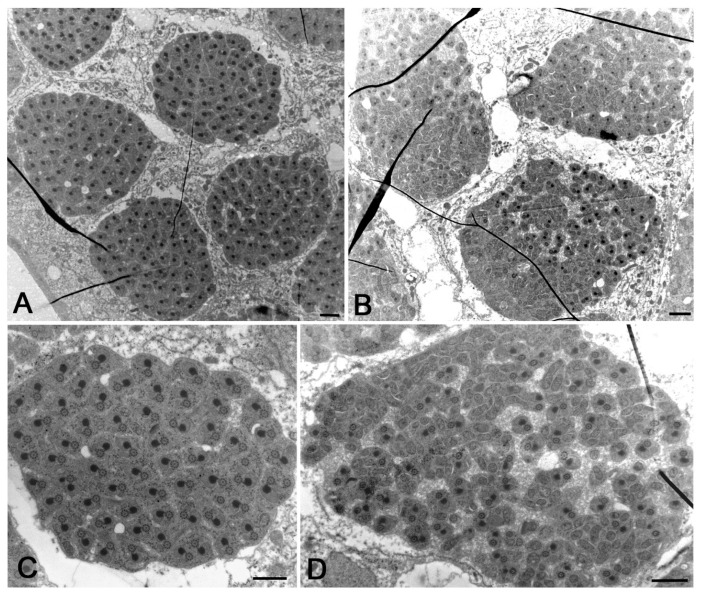
Low magnification of uninfected or poorly infected (**A**) and highly infected (**B**) cysts of mid-elongating spermatids in testes of late DSR pupae. (**C**) Uninfected spermatids are thin and closely packed. (**D**) Infected cysts contained spermatids with a large cytoplasm filled by bacteria and thin spermatids without bacteria; the spermatids are poorly packed and large empty spaces are seen among them. Scale bars: (**A–D**), 2 μm.

The bacteria inside the elongating spermatids dramatically affected the shape and the structure of the whole cysts (Figure 4A,B). Mid-elongating spermatids devoid of or with isolated bacteria had a small diameter and were closely apposed within the cyst without large extracellular spaces among them (Figure 4C). Conversely, mid-elongating spermatids holding high bacteria densities showed variable dimensions depending by the number of bacteria hosted within the cells (Figure 4D). Moreover, most of the spermatids within the infected cysts were separated by empty spaces (Figure 4B,D).

**Figure 5:**
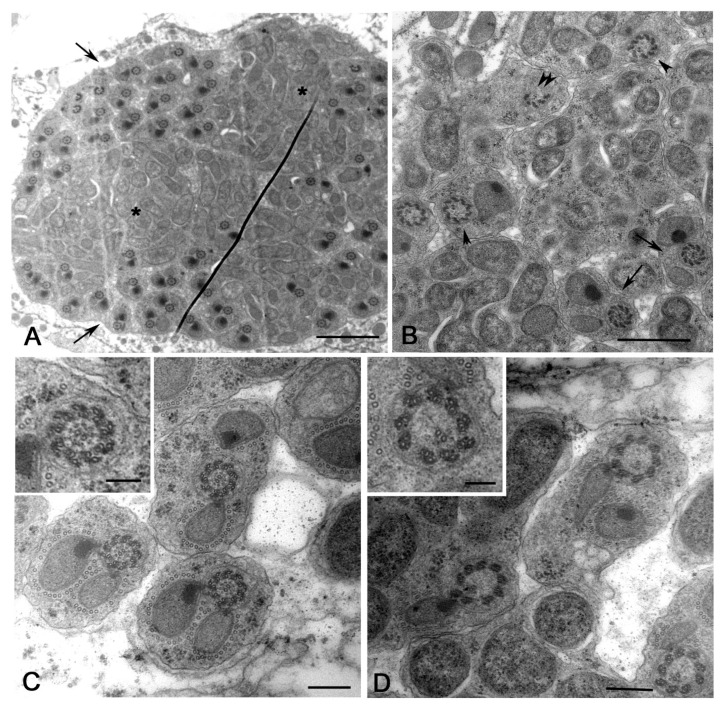
Cross-sections of the distal region of late-elongating spermatids in testes of late DSR pupae. (**A**) Whole infected cysts showing a reduced number of axonemes (arrows) and many bacteria (asterisks). (**B**) Detail of an infected cyst consisting of normal-looking axonemes with a pair of mitochondrial derivatives (arrows), larger axonemes with incomplete set of mitochondria (arrowheads), and fragmented axoneme (double arrowheads). Details of spermatids with normal (**C**) and abnormal (D) axonemes. The microtubule wall of the abnormal axonemes (inset, **D**) are larger than the normal ones (inset, **C**); moreover, the central tubules of the abnormal axonemes are offset from the centre and the radial spokes are missing (inset, **D**), whereas the normal axonemes have central tubules in the proper position and distinct radial spokes (inset, **C**). Scale bars: (**A**), 2 μm; (**B**), 1 μm; (**C**,**D**), 200 nm; insets, 100 nm.

Cross-sections through the distal region of late-elongating spermatids that contained a high bacterial density (Figure 5A) showed the most dramatic structural alterations of axonemes. We find, indeed, cysts containing spermatids either with normal-looking axonemes (66% of spermatids, *n* = 237 spermatids examined from seven cysts) or abnormal axonemes showing assembly or maintenance defects (34% of spermatids, *n* = 237 spermatids examined from seven cysts) (Figure 5A,B). 

Normal-looking axonemes consisted of the usual nine outer doublet microtubules, one pair of central microtubules, and nine peripheral accessory fibers (Figure 5C). Nine radial spokes were evident between the central pair and the peripheral doublets (Figure 5C, inset). Conversely, spermatids at the margin of the cysts displayed axonemes in which the central pair was misplaced, and the peripheral tubules lost their continuity and lay on large elliptical walls (Figure 5D). Incomplete axonemes lacking some peripheral tubules and the central tubule pairs were also observed (Figure 5B). The radial spokes were no longer found inside the abnormal axonemes (Figure 5D, inset). 

### 3.4. Ciliary Cap Defects in Elongating Spermatids of Late DSR Pupae

**Figure 6:**
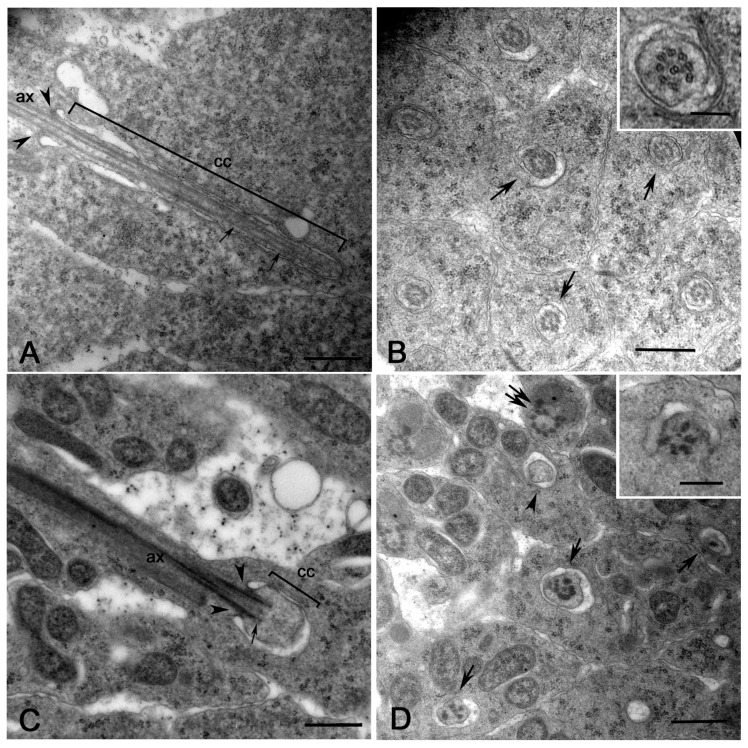
The ciliary cap of the growing axoneme in late DSR pupae. (**A**) Longitudinal section of a normal ciliary cap (cc, bracket); arrowheads point to the transition region between the spermatid cytoplasm and the ciliary cap. Small arrows point to microtubules inside the ciliary cap: ax, axoneme. (**B**) Cross-sections of ciliary caps (arrows) showing microtubule doublets that organize the 9 + 2 axoneme; detail of a ciliary cap (inset, **B**) showing nine microtubule doublets and the C-remnant associated with the B-tubule, but the central pair is absent in these sections. (**C**) Longitudinal section of an abnormal ciliary cap (cc, bracket) showing the reduced extent of the microtubules inside the cap (small arrow); arrowheads point to the transition region between the spermatid cytoplasm and the ciliary cap: ax, axoneme. (**D**) Cross-sections of abnormal ciliary caps containing dense scattered tubules (arrows) or empty cytoplasm (arrowhead); double arrows point to an abnormal axoneme. Detail of the transition region between the cytoplasm and the ciliary cap (inset, **D**) showing a few dense tubules. Scale bars: (**A–D**), 500 nm; insets, 100 nm.

The distal tip of the growing axoneme in elongating spermatids was surrounded by a distinct plasma membrane, giving rise to a compatmentalized region, the ciliary cap, that extended 4–5 μm over the posterior end of the spermatids (Figure 6A, bracket). Here, the microtubules (Figure 6A, small arrows) assembled and organized in a regular pattern to form the cytoplasmic axoneme. A narrow region at the transition between the cell cytoplasm and the ciliary region physically separates the two cytoplasmic compartments (Figure 6A, arrowheads).

Cross-sections at the distal end of the ciliary cap showed microtubule doublets or single microtubules arranged in incomplete circular arrays (Figure 6B, arrows, and inset). This disposition points to the initial steps of the axoneme assembly which take place in the ciliary cap. By contrast, some spermatids (29% of spermatids, *n* = 87 spermatids examined from four cysts) at the margin of the cysts had shorter ciliary caps that enclosed a reduced axonemal tip (Figure 6C, bracket). Cross-sections of the short ciliary caps showed few dense tubules (Figure 6D, arrows), pointing to the failure of the proper axoneme assembly. This is supported by the sections 1–2 μm away from the base of the ciliary cap that only revealed empty cytoplasm (Figure 6D, arrowhead). The short ciliary caps could be retained at the distal ends of the abnormal elongating axonemes (Figure 5B,D). This is supported by sections of the transition region between the cytoplasm and the short ciliary caps that showed few dense tubules arranged in a disorderly fashion (Figure 6D, inset). Moreover, the irregular axonemes were observed in the same cysts also containing the short ciliary caps (Figure 6D, double arrows). 

### 3.5. Failures of the Individualization Process in Young Adult DSR Males

The spermatids that reached their full length underwent a final differentiation process, individualization, consisting of the elimination of a large amount of cytoplasm and organelles to make the sperm thin and able to move. This process was driven by peculiar actin rich structures, the actin cones, that emerge from the posterior region of the nucleus and run alongside the axoneme until the posterior end of the tail [36,37]. During their movement, the actin cones push the excessive cytoplasm in distinct cyst bulges and then in waste bags at the terminal end of the sperm [38]. Bacteria, also, were gradually pushed by the actin cones into the terminal end of the sperm and collected in the waste bags. Thus, the cytoplasm of elongated spermatids filled with *Wolbachia* was cleared during the individualization process to give rise to tiny sperm devoid of bacteria [29].

**Figure 7:**
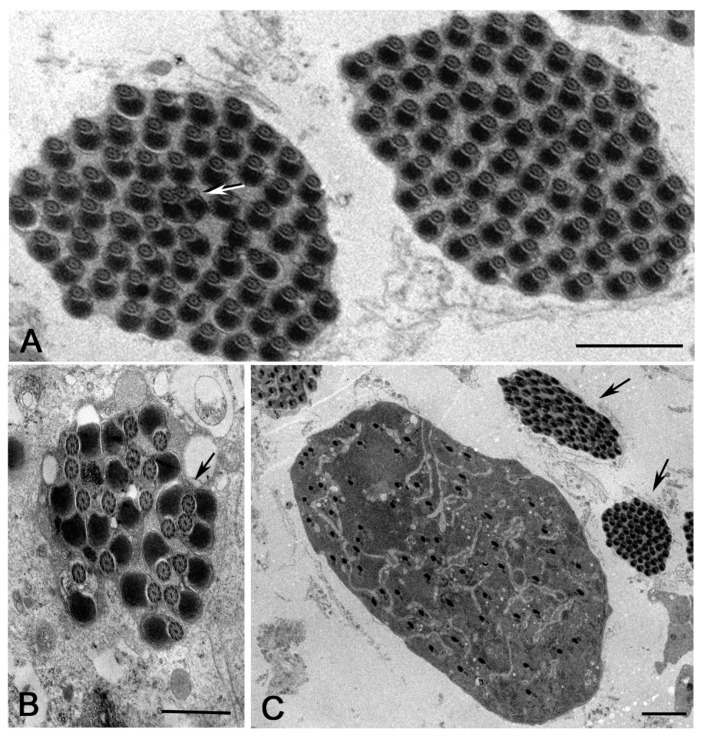
Cross-sections of whole mature sperm cysts from testes of young DSR males. (**A**) The individualization process leads to compact clusters of 64 sperm each consisting of a 9 + 9 + 2 axoneme and a dark mitochondrial derivative; usually, the mitochondria have the same orientation within the cysts (**A**, right cyst), but there are also cysts in which the mitochondria lost their regular orientation (**A**, left cyst); sperm with two axonemes and two mitochondria are also found (arrow). (**B**) Cross-section of a cyst containing an incomplete number of sperm; arrow points to sperm with three axonemes. (**C**) When the individualization process fails, the cysts contains axonemes interspersed within large cytoplasmic areas; moreover, these abnormal cysts appear larger than the properly individualized cysts (arrows). Scale bars: (**A**), 2 μm; (**B**), 1 μm; (**C**), 3 μm.

The individualization process leads to cysts of 64 closely packed haploid sperm, consisting of a thin cytoplasm containing the 9 + 9 + 2 axonemes and condensed mitochondrial derivatives (Figure 7A). The complex axoneme–mitochondrial derivatives approximately display the same orientation in 38% of cysts (*n* = 127 cysts examined) (Figure 7A). We also find cysts in which this complex is randomly oriented (33% of cysts, *n* = 127 cysts examined) (Figure 7A). Moreover, some sperm in the same cyst sometimes displayed two or more axonemes (22% of sperm, *n* = 632 sperm examined from 19 cysts) (Figure 7A). In addition to cysts with 64 normal-looking mature sperm, some cysts with remarkably numerical and structural sperm abnormalities have also been observed. We find, indeed, cysts showing an incomplete number of mature sperm (16% of cysts, *n* = 127 cysts examined) (Figure 7B). The individualization process was improperly performed in some cysts (13% of cysts, *n* = 127 cysts examined) and the sperm were dispersed in large cytoplasmic areas (Figure 7C). The incompletely individualized sperm cell cysts appeared larger than the fully individualized cysts (Figure 7C). 

### 3.6. Abnormalities of the Individualized Sperm in Young Adult DSR Males

**Figure 8:**
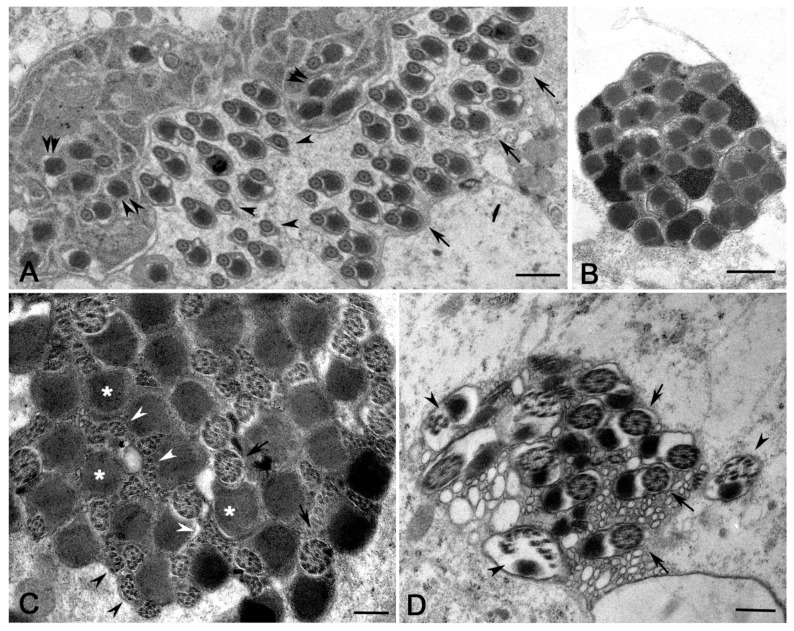
Structural defects of mature sperm in testes of young DSR males. (**A**) Cross-section of a defective individualized cyst containing normal mature sperm (arrows), mature sperm with only the axoneme (arrowheads), and sperm with only the mitochondrial derivative (double arrowheads). (**B**) Whole cyst in which all the sperm tails lack axonemes and only the mitochondrial derivatives are present. (**C**) Cross-section of a cyst containing normal-looking axonemes (arrows) and several free doublets (arrowheads) interspersed between dense mitochondria (asterisks). (**D**) Detail of a cyst with a reduced number of sperm: some of the sperm have a normal axoneme (arrows), whereas others have a fragmented axoneme with disorganized dense tubules (arrowheads). Scale bars: (**A**,**B**), 1 μm; (**C**), 400 nm; (**D**), 500 nm.

Improperly individualized cysts may contain normal mature sperm along with abnormal sperm that lack mitochondria or axonemes (Figure 8A). Remarkably, we also find whole clusters of sperm tails lacking the axoneme and only consisting of condensed mitochondrial derivatives (Figure 8B). Some individualized cysts showed normal-looking axonemes, along with axonemes that retained the nine peripheral doublets but lost the nine-fold symmetry and appeared deformed (Figure 8C). Several doublets were also interspersed between the mitochondrial derivatives (Figure 8C). Individualized cysts containing reduced numbers of mature sperm may also include sperm with fragmented axonemes (Figure 8D). In none of the cysts consisting of normal or abnormal individualized sperm did we observe bacteria. 

## 4. Discussion

The study herein extends previous conventional immunofluorescence observations regarding the distribution of *Wolbachia* during the spermiogenesis of *DSR*-infected males [29,30,39,40], giving new information on the impact of bacterial infection on sperm maturation. 

Testes of late pupae and young 3–5-day-old males from *Wolbachia*-infected *DSR* contain cysts with normal germ cells and cysts with germ cells showing a great variety of defects. Remarkably, normal-looking cysts often grouped together in clusters spatially separated by the cysts containing abnormal germ cells. 

An ultrastructural analysis of pre-individualized spermatids revealed three major defects: abnormalities of the mitochondria, structural defects of the axoneme and failure in achieving its full elongation, and incomplete number of germ cells within the cysts. Such defects are highlighted in abnormal mature sperm, which also suffer from incomplete individualization. 

The defects in maintaining the proper axonemal structure become evident in the distal regions of most spermatid cysts harbouring a high bacterial density. The radial spokes of the axoneme are no longer detectable and the central tubule pairs lose their usual position at the center of the axoneme. Concurrently, the nine peripheral doublets lose all links and move slightly away from each other. Thus, the axonemes increase their diameter and lose their circular arrangement. Axonemes with incomplete numbers of peripheral tubules have been also observed. These findings suggest that the central tubule pair, besides ensuring the proper sperm beating, may play a main role in maintaining the structural integrity of the ninefold axoneme through the radial spokes. 

The axonemes with a defective disposition of the peripheral doublets fail to fully elongate, as inferred by the reduced size of the distal ciliary caps where the axonemal microtubules would assemble to warrant the proper growth of this structure. The ciliary cap represents a compartmentalized domain essential for the integrity of the axoneme assembly [41]. The entry of the axonemal precursors into this structure occurs through the ciliary gate, a specialized region at the basis of the ciliary cap [41]. Moreover, axonemal proteins are added to the bare microtubules as they are displaced from the ciliary cap into the cytoplasm [42]. It can be hypothesized that the presence of a large number of bacteria near the ciliary gate could hamper the free transit of the molecules necessary for the elongation and maturation of the axoneme.

All the abnormal axonemes consist of microtubules filled by dense material. It is difficult to determine whether it is the dense material that causes the degradation of the axoneme or whether it is the degeneration of the axoneme that leads to the accumulation of dense material. However, whether the presence of *Wolbachia* is related to this material is unclear.

Young spermatids have many scattered mitochondria that fuse together in a single onion-shape structure, the nebenkern. The nebenkern soon unfolds into two mitochondrial derivatives that elongate in association with the growing axoneme [43]. The size and shape of the mitochondrial derivatives and their orientation are not uniform within the infected cysts. The elongation of the mitochondrial derivatives and, presumably, their stability and maintenance are correlated with the longitudinal microtubule bundles that emerge from the centrosomal material surrounding the sperm basal body [44]. Since the spermatids with abnormal mitochondria show few randomly arranged longitudinal microtubules, it is conceivable that the bacteria could disturb the proper arrangement of the longitudinal microtubules, thus affecting the correct dynamics of the mitochondria.

The mitochondrial derivatives, together with the longitudinal bundles of cytoplasmic microtubules, are retained to support spermatid elongation in *Drosophila melanogaster* [44,45], and the shape of the mitochondria is also important for the correct individualization of the *Drosophila* spermatids. It has been shown that the abnormal size of the mitochondria could influence the progression of the individualization complexes leading to the failure of sperm maturation [46,47,48]. However, the abnormalities in shape and size of the mitochondria found in the infected testes of *DSR* seem too mild to fully explain the dramatic individualization defects observed. 

Therefore, an important question here is how the structural alterations observed in elongating spermatids and individualized sperm of *DSR* could be correlated with the presence of *Wolbachia*. A first explanation could be that unknown factors released by the bacteria could affect the proper morphogenesis of the germ cells. According to this possibility, it has recently been suggested that *Wolbachia*-induced metabolic changes lead to feeble defects of the mitochondrial derivatives in *Drosophila melanogaster* [31]. However, the great heterogenic variety of the ultrastructural defects associated with the spermatid elongation in infected testes are unlikely to be exclusively the result of bacterial by-products. Rather, these defects could be explained as downstream effects of the increased concentration of bacteria within the germ cell cytoplasm that could represent a spatial obstacle for the normal dynamics of the spermatids. *Wolbachia* density could impose a physical constraint that forces mitochondria and axonemes to bend during their linear extension. Failure to grow in a straight direction to avoid bacterial obstruction can result in pressing lateral forces that impact the mitochondrial morphology and axonemal structure. Because the elongation of mitochondria and axonemes usually occurs in parallel along the spermatids of the same cysts, any abnormality could also interfere with the normal dynamic of adjacent cells. 

The bacteria, when present in high concentrations in the cytoplasm of the spermatids, can also cause spatial hindrance that alters the synchronous movement of the actin cones towards the posterior end of the germ cells, with the consequent failure to push the excessive cytoplasm and organelles in the distal waste bag. The findings of cysts with mature sperm interspersed within large cytoplasmic areas suggest that the individualization complexes may encounter serious difficulties in moving the large clusters of bacteria inhabiting the germ cells. Thus, the individualization process could be incompletely performed in the presence of a high bacterial density.

The low number of spermatids present in several abnormal cysts points to earlier defects in the meiotic progression. Therefore, *Wolbachia* could also play a role during spermatogenesis by altering the meiotic divisions, perhaps interfering with the organization of the spindle microtubule. It has been reported, indeed, that *Wolbachia* can move along microtubule tracks [49,50,51] and, if present in a high concentration, could disturb the normal dynamics of the meiotic spindles. We also find cysts in which the structural defects are restricted to a few spermatids. This observation raises questions about the functionality of the apparently normal sperm cells, since the germ cells of each cyst are interconnected together by cytoplasmic bridges and, presumably, communicate among them. Thus, defects in some spermatids could compromise the proper dynamics of the whole cyst. However, with the disruption of the cytoplasmic bridges following individualization, the normal sperm would be completely separated from the modified ones, which would be eliminated. In this way, the abnormal cysts could contribute some functioning sperm.

It has been shown that 3-day-old and 5-day-old infected males produce approximately 62% and 57% as many sperm as uninfected males [22]. The ultrastructural alterations observed in randomly made cross-sections of elongating spermatids and mature sperm (DSR: 87% abnormal germ cells, 869 germ cells examined from 23 cysts; DSW: 01% abnormal germ cells, 732 germ cells examined from 19 cysts) well explain the reduced fertility of infected males compared to their uninfected counterparts.

The grossly disorganized sperm cysts seen in infected males of *DSR* may, in an unlikely way, be connected to cytoplasmic incompatibility, because the structurally modified sperm is presumably nonfunctional and, therefore, may not be transmitted to the females and, ultimately, is unable to fertilize the eggs. Therefore, a high *Wolbachia* density is harmful for proper sperm differentiation and function. Thus, the impact of *Wolbachia* on *DSR* spermiogenesis could result in a decrease of functional sperm production. High bacteria numbers can also lead to a reduced lifespan in flies [21], suggesting that optimum *Wolbachia* densities are important for a stable endosymbiosis [52,53]. 

These observations open questions about the relationship between the presence of *Wolbachia* and sperm integrity, suggesting that the functional sperm released by infected males and capable of contributing to cytoplasmic incompatibility must mainly derive from spermatocytes lacking bacteria or with low bacteria densities. An optical microscope analysis of the spermatogenesis in *DSR*-infected males reveal, indeed, the presence of highly or partially infected spermatocytes, together with spermatocytes without bacteria [29,30]. However, the number of the cysts containing uninfected germ cells is much lower than the number of infected cysts. 

## 5. Conclusions

The ultrastructural data obtained here show that the presence of the alphaproteobacteria *Wolbachia* in the testes of the *DSR* strain is associated with a plethora of defects highlighted during the early and late stages of spermiogenesis. The main defects observed were structural and numerical alterations of the mitochondria, structural abnormalities of the axonemes and failure to fully elongate, supernumerary axonemes within the same germ cell, an incomplete number of spermatids, and an incomplete individualization process.

Further analyses are needed to clarify the involvement of the Cifa and Cifb proteins during the complex mechanisms that ensure correct spermatid elongation and sperm maturation.

## Data Availability

Not applicable.
